# Discovering Students’ Personalised Uses of a Paediatrics Acute Care E-book – a Qualitative study

**DOI:** 10.15694/mep.2019.000189.1

**Published:** 2019-10-09

**Authors:** Toby Price, Peter Thomas Cartledge

**Affiliations:** 1 University of Leeds

**Keywords:** Medical students, Medical education, Teaching materials, Textbooks, Multimedia

## Abstract

This article was migrated. The article was marked as recommended.

**Background:** Medical educators are increasingly developing and recommending e-books to supplement students’ learning. Despite this, there is a gap in the literature surrounding E-book developers’ understanding of how students can personalize an e-book for multiple different purposes. This qualitative research project aimed to explore students’ experiences of using a pediatric acute care E-book (pRRAPID) within a spiral, multi-method teaching package. The researchers aimed to get an insight into the ways that learners used E-books outside of the classroom.

**Methods:** Three focus groups were conducted with 12 fourth-year medical students who had completed a pediatric acute care course. Students were invited to discuss their use of the E-book during the course. Thematic analysis was conducted on the transcripts to identify codes in the data; these were grouped to determine the overarching themes.

**Results:** Students outlined five different ways that they personalized the use of the E-book. Three of these were related to uses of the E-book within the context of the pediatric acute care course; students using the E-book to prepare for a formal teaching session (“Flipped classroom“),students using the E-book as a workbook (going from start to end) and students using the E-book to prepare for an exam. The other two themes related to the use of the E-book outside of the acute care course; students using the E-book as a resource “on the go” and students using the E-book within clinical practice.

**Conclusions** This qualitative study outlines how an educational resource can be utilized in numerous different ways in order to satisfy the needs of the student. Focus-group participants outlined how important it was to engage with the students, as the primary stakeholder, when developing an E-book.

## Introduction

An electronic book (or E-book) is a book made available in digital form, consisting of text, images, or both and readable on smartphones, tablets, computers or electronic readers. Literature suggests that students using E-books perform as well as those using traditional physical (hard) copies of the text ( Stirling and Birt, 2014). However, there seems to be disagreement about whether they are a time-efficient way of learning ( Shepperd, Grace and Koch, 2008; Daniel and Woody, 2013). Some studies have suggested that some readers may perceive that E-books are harder to read than physical (hard) paper books ( Van der Velde and Ernst, 2009; Franziska *et al.*, 2013; Kim *et al.*, 2014).

Reported advantages of E-books are decreased cost ( Bialaszewski, 2013) and the practicality of being more transportable and accessible. Furthermore, E-books appear to integrate the principles of “scannability” to a greater extent; “scannability” refers to the utilization of techniques such as headings, bulleted lists, graphics, etc. to aid quick reading and understanding of a piece of writing ( Morkes and Nielsen, 1997). A major advantage of E-books is that they aid navigation within the text. Navigation within an E-book is fundamental to increasing the usability; having an interactive contents page, links between pages and clear titles have improved user experience in a previous study ( Chong, Lim and Ling, 2009). E-books ensure that all students have materials available for study, rather than just those who have the financial or practical means to purchase a physical book or access it from a library. Access to relevant materials is important for curricula that use a “flipped classroom” asking students to pre-read material before attending teaching sessions ( Eaton, 2017).

Studies have shown that students are supportive of the implementation of E-books into educational practice in order to supplement rather than replace current practice ( Pickering, 2015). Further research is required in order to gain an understanding of the interaction between the student and an E-book; this would enable a greater understanding of factors that enable students to maximize a resource’s capability.

Previous technology-enhanced learning research identified that students could use the same resource in a variety of different ways. The authors of this work termed this “personalization” ( Joynes and Fuller, 2016). Personalisation is the idea that the user and not the developer determine the purpose of an E-book. This takes into consideration some models for adaptive learning such as the Dunn and Dunn model which assess the most effective learning style for the students in question; these draw upon ideas like visual, auditory and kinesthetic learning ( Lovelace, 2005). As a result, early stakeholder involvement in E-book production is essential to determine individuals’ ideas about the purpose of an E-book and hence design the resource with these ideas in mind ( Joynes and Fuller, 2016). This echoes the current switch within medical education, where there is a shift towards emphasizing learners’ responsibility for their own learning ( Mann, 2011).

In the literature, there is a significant emphasis on the product developer assessing the learning methods of the users to “personalize” the product ( Klašnja-Milićević *et al.*, 2011; Essalmia *et al.*, 2015; Bourkoukou and El Bachari, 2016). However, this emphasis assumes that learners’ preferences are stable, though it is more likely that students use different learning strategies at different times ( Lujan and DiCarlo, 2006). Furthermore, the emphasis on personalization at the time of use detracts from the potential role of students in determining the application of an E-Book during the development phase. There is limited literature about students determining the use of an E-Book, and it appears that the E-book developer then the user more often determines the application of the resource. This is an interesting observation when it has been seen that students who have autonomy over their use of e-Learning are more motivated to engage ( Huang, 2008).

There has been increased levels and access to technology and a greater emphasis on students’ responsibility for their own learning ( Alur, Fatima and Joseph, 2002). Due to the relatively recent rise of E-books and diversity in their implementation, there is a lack of comprehensive research to test their effectiveness or to explain how students use E-books to aid their personal learning.

***Research context:*** The “Recognising and Responding to Acute Patient Illness and Deterioration” (RRAPID) course ( http://rrapid.leeds.ac.uk/) is a spiral curriculum that runs throughout the University of Leeds undergraduate medical course. The primary teaching method is simulation training, supported by reading materials for the theoretical background. Pediatric RRAPID (pRRAPID) has now been added to this spiral curriculum in order to equip students with parallel skills within pediatrics. pRRAPID is a package of learning offered through a combination of; a peer-taught symposium, immersive simulation training, small group prescribing teaching and an E-book.

The E-book contains theoretical background, questions to aid knowledge acquisition and scenarios to demonstrate the application of knowledge. The E-book was created to give the students the theoretical information they needed to prepare and follow-up on the peer-taught symposium and simulation sessions. The PI and second author were both directly involved in developing the E-book along with a team of senior pediatricians, speciality trainees (residents), nurses, resuscitation officers, a pharmacist and students (E-book users).

***Research Objectives:*** This research project aimed to explore students’ experiences of using this E-book within a spiral, multi-method teaching package, in order to get an insight into the personal interaction between learners and technology. Two research questions were explored: How do medical students use an E-book and what are students’ views on the strengths and weaknesses of an E-book? During the analysis, themes of motivation and personalization developed; therefore, a new research question developed exploring how students use of the E-book developed student motivation and how they personalized the resource.

## Methods

**Study design:** This was design-based research which employed qualitative methods (The-Design-Based-Researcher-Collective, 2003). Reporting of this qualitative study has been verified in accordance with the COREQ and SRQR checklists for qualitative studies ( Tong, Sainsbury and Craig, 2007; O’Brien *et al.*, 2014).

**Qualitative approach:** The research was undertaken within an interpretivist approach, as the researcher was aiming to understand students’ individual experiences with the E-book rather than measuring the effectiveness of the E-book ( Bunniss and Kelly, 2010). Qualitative methods were chosen as it was agreed that quantitative survey data would not gain the richness of data required to explore the research questions.

**Researcher:** The principal investigator (PI) was involved in the production of the E-Book and was interested in finding out whether people had used the resource and if so, how they had used the resource. The research was undertaken for the purposes of his intercalated medical education degree and for learning how to apply qualitative methods. The PI was supervised by an academic experienced in qualitative research.

**Participants:** Fourth-year medical students who had taken part in the pRRAPID teaching in the previous week, who would still be able to recall their experience of the E-book were eligible for inclusion. Exclusion criteria were; students who had completed pRRAPID before ethical approval. *Non-participation:* Of the 240 students in the year group 125 of these took part in the pRRAPID course. Seventy-five students were eligible after gaining ethical approval. Of these 12 students responded to the invitation.

**Sampling:** Purposive sampling was employed, to identify fourth-year medical students who had taken part in the pRRAPID course. Potential participants were asked to indicate their availability for attending a focus-group and the most popular times were selected. Participation was therefore limited to those who could attend at the selected times. All students in the year group were contacted by email, with an explanation of the study and the consent form to allow potential participants the opportunity to consider taking part. The PI was a member of the year group below the participants and therefore verbally disseminated requests to support the email invitation. Some of the participants were known to the PI; the effect of this was minimised by having a robust informed consent procedure, conducting the interviews in university and using a pre-designed interview question guide.

**Focus groups:** Three focus groups were employed. Focus-groups were used to encourage respondents to explore and clarify individual and shared perspectives ( Tong, Sainsbury and Craig, 2007). This was seen as optimal in comparison to individual interviews because we were interested in hearing multiple perspectives from the group who undertook the pRRAPID course and focus-groups provide a greater depth of data, as they allow elaboration on interesting points ( Hanson, Balmer and Giardino, 2011).

ThePI led the focus groups. They started with an “ice-breaker” question and then were conducted using pre-planned open-ended questions. A conversation then ensued between the members of the focus-group, facilitated with further questioning. The interviews were based on a focus-group guide. Flexibility in the order of questions was used to allow each of the focus-groups to follow the direction that the participants took it; however, allowed a general structure to keep the participants talking about subjects relevant to the research topic. This focus-group guide was developed in accordance with one of the project supervisors who was not involved in the production of the e-Book. Henceforth, it was important to follow the guide as it helped to reduce the level of bias that may have been present by the Participants were then given the opportunity to raise any final thoughts about the E-book that may have been missed.

### Question Outline for Focus-groups

• How was your pediatric placement? (ice-breaker)
• Tell me about your experience of using the E-book?
  • How often?
  • When?
  • Where?
  • For what purpose?
• Were there particular times/parts of the course when the E-book was more useful?
  • Would you have managed to complete the course without the E-book?
  • How did you use the ebook in preparation for the student-led sessions?
• Did you use the scenarios?
  • What would make you more likely to use them?
  • What would make them better?
• What content was essential?
• What content was less essential?
• What would make you use the E-book more?
  • What can be improved for next year?
  • How important is it that the material is electronic?
• Would you prefer the ebook in app form?

Each focus group lasted approximately one hour. Field notes were not taken during the interview. The focus groups were recorded using an electronic dictaphone; audio files were then transferred immediately onto secure university computers and anonymized and transcribed verbatim by the Principal Investigator (TP).

**Content Analysis:** Transcripts were read repeatedly to ensure familiarisation. Thematic analysis of the transcripts was undertaken to derive meaning from the data, drawing on guidance from Braun and Clark (2006). The data were coded and analyzed after the first focus-group in order that the results could inform questions asked in the subsequent focus-group. Codes were usually one or two words long ( Erlingsson and Brysiewicz, 2017). Quotes for each code were then grouped to allow interpretation of the themes. Initial coding was performed by the PI as the interviews progressed. Final transcripts were cross-checked and coding of each quote reviewed by the second author (PC) and the project supervisor (AL). This multiple levels of review was important in reducing the level of bias resulting from the PI being personally known to the participants.

Themes were developed by grouping the codes whereby a theme was seen as expressing an underlying meaning ( Erlingsson and Brysiewicz, 2017). The themes were then reviewed and refined through discussion between the authors.

## Results/Analysis

**Participants:** A total of twelve participants took part in the three focus-groups, five in the first focus-group, three in the second focus-group and four in the final focus-group. All of the participants were between the ages of 23 to 25-years-of-age. Six of the twelve participants were female.

**Figure 1.**
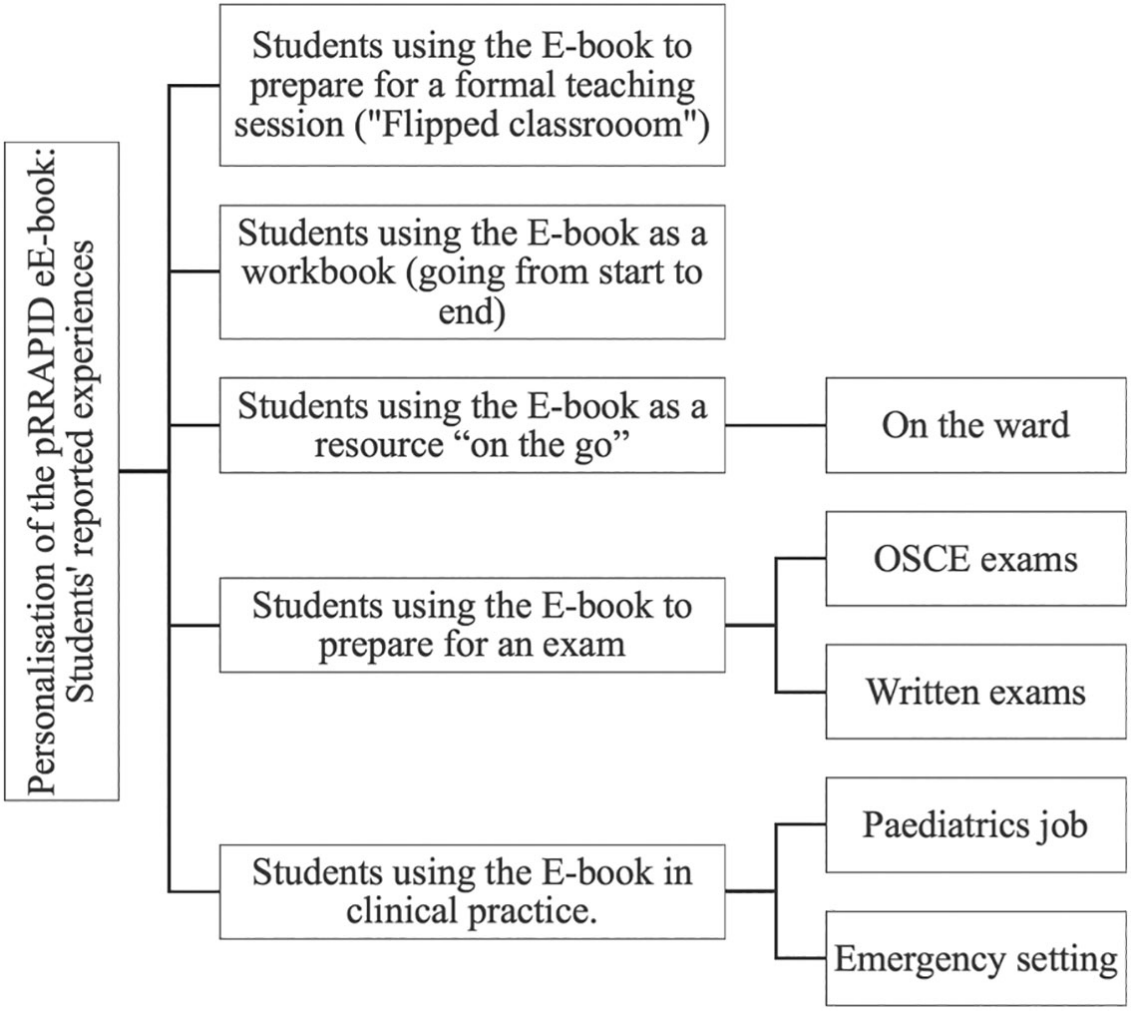
Themes and subthemes of E-book use

Within the focus groups, students discussed the strengths of the pRRAPID E-book and suggestions for improvement of E-books. However, most interesting were the students’ accounts of the various ways they had used the E-books to support their learning. Here we explain five themes related to personalization of the pediatric acute care e-book ( Figure 1).

**Students using the E-book to prepare for a formal teaching session (“Flipped classroom”):** Within the pRRAPID course, students were required to attend three interactive sessions which included activities such as peer-teaching, small group workshops, and simulation. One participant in the focus-group outlined that they had completed some of the activities in the E-book before attending the prescribing session so that they could use the session to check on their knowledge and understanding.

*“It was good to practice the prescribing (..) we were told to do it beforehand so (..) when we got there, and it was sort of like checking through” (FGP7)*

Participants in the study outlined that doing work before a session was supportive in learning the content because of the extent of repetition between the prior work and the session.

*“If you go straight into a session without doing it or having any practice, I don’t really like doing that, and I don’t really get much out of it” (FGP9)*

On the contrary, some participants outlined that they felt that using the E-book before a session was time-consuming, created uneven starting points in the session and they were frustrated by the repetition between the prior work and the work in the session.

*“For me, it felt kind of pointless to turn up and go through it when the answers were already on the book. So, I had already gone through, tried it, checked my answers” (FGP7)*

*“I don’t think you necessarily have time to do exercises before every session, so I am not entirely convinced that everybody would have done it. And then you are all kind of starting from an uneven start point. So some people will understand most of how to prescribe and some people will be at the baseline” (FGP11)*

**Students using the E-book as a workbook (going from start to end):** Participants described using the E-book as a workbook, working through the whole book from top to bottom.

*“The fact at the end of each section there was a scenario that you could answer the questions and go down and work through it. Rather than just reading the whole document and actually spend hours doing it which made you learn it rather than just passively reading it. Which I thought was really really good.” (FGP2)*

*“So we had a drugs chart and a fluid chart that we got given at the beginning so that as you were working through each of the scenarios, you (..) put it either on the drugs chart or the fluids chart. Which is quite useful actually, I think because there was quite a lot of things that needed (..) it was all the repetition, doing a thing, again and again, that really brought that home for me” (FGP1)*

This stimulated an interesting conversation with another individual in the same focus-group who initially felt this was not the way that he preferred to learn. However, at a later point in the focus group, he indicated that he would go through the whole E-book before he completed their exams.

*“Different people learn differently, so obviously [Candidate 2] would rather have the whole text in front of him and work through it, but some people would probably find that a daunting thing to look at and having it broken down would be a better way for them to learn it” (FGP1)*

*“Certainly before I get to the OSCEs I want to have gone through it all at least once and gone through the cases because I think I will get a lot out of that” (FGP1)*

**Students using the E-book as a resource “on the go.”:** The original intention for the E-book was that it would be used whilst “on the go” to provide learning wherever the students would be. It was then interesting to see that the participants did not feel that they could use the E-book like this both because of the size and the barriers to using mobile phones on the wards.

*“If you are sat there looking at your phone on the CAT [children’s assessment and triage] unit that would be dodgy, and everybody would be like “what’s this joker doing?” I’m not exactly going to sit there reading something off my phone” (FGP6)*

**Students using the E-book to prepare for an exam:** Many of the participants outlined that they intended to use the resource to prepare themselves for their exams despite the resource not being designed as a revision tool for University of Leeds examinations.

*“It highlights the key skills for OSCEs, like the way that it comes in the SBAR [situation, background, assessment, recommendation] form, and then you go on, and at the end, there is the prescribing of whatever” (FGP4)*

*“I know that we aren’t going to be tested on pRRAPID, but I am still going to go through the pRRAPID book again because of it’s usefulness and relevance to everything else on paeds” (FGP2)*

**Students using the E-book to prepare for future clinical work:** Participants in the study outlined that they might use this resource to prepare for employment as a clinician in pediatrics or Accident & Emergency despite the fact that the content of the E-book was intended to be at the level of a medical student.

*“Do you think you will flick back to it after fourth year?” (Interviewer)*

*“If you came to doing a pediatrics job you probably would” (FGP4)*

*“Or an A&E job” (FGP5). “Probably would” (FGP2)*

However, it didn’t come as a surprise that students felt that they couldn’t use the resource in an emergency situation due to the inaccessibility of the information when in a rush.

*“I think that you won’t ever be in a pRRAPID situation and think “oh I need to click on the pRRAPID app for this bit of information” because you just won’t get that” (FGP2)*

## Discussion

This research project aimed to establish how different students were using the pRRAPID E-book; the research team aimed to get an insight into how the students used the same resource for different purposes. This discussion aims to analyze the different uses of the E-book and to compare it with previous literature.

Previous research surrounding E-books has focused on the effect of E-books on examination scores ( Shepperd, Grace and Koch, 2008; Daniel and Woody, 2013; Stirling and Birt, 2014), hence the idea that an E-book could be used to prepare for an exam was not a surprising finding. In this study, the students perceived that the E-book would be helpful in preparation for the OSCE and written exam and gave them confidence that they had read everything they needed to know. These findings were aligned with the findings from Woltering *et al*. ( Woltering *et al.*, 2009) where students who used an E-book felt that they had higher perceived learning gains. Furthermore, the idea that students could use an E-book to prepare for a teaching session was not a new one; Stirling and Birt ( Stirling and Birt, 2014) found that students’ attainment after an anatomy session was higher when they had used an E-book in preparation for their session. In our participants, students outlined that they “ *used it before that prescribing session”* (FGP4) and they “ *used it for information to talk about.. with the slides [in the peer teaching session]”* (FGP8).

It was observed that the E-book was fulfilling two different purposes: as a helpful reference book and a workbook. One participant emphasized its use as a reference book, saying “ *it is good to have everything in one place, so if you wanted to learn anything about pRRAPID you could go to this book.*” This was in line with the research by Gardner *et al.* who found that it was important to students that there was a centralized, reliable source of information to learn from. The second use of the E-book was “ *as a workbook”* (FGP6). These different usages mirrored the work by Joynes and Fuller about personalization who explained how users of technology themselves define the purpose of E-learning ( Gardner *et al.*, 2016; Joynes and Fuller, 2016). In educational literature more generally, it is also recognized that learning can deviate from the intended curriculum ( Hume and Coll, 2010). Focus-group responses indicated that students were using the interactive questions in addition to the block text; participants stated these questions increased engagement with the E-book and subjectively helped them to consolidate their knowledge.

The potential use of E-books for later pediatric practice was a surprising finding, as this was beyond the intended use of the resource. However, there is literature to suggest that doctors could use such a resource in the workplace. Nolan (2013) reported that access to technology in the workplace has already been established with 82% of doctors currently owning a smartphone, this being in 2011 and more likely to be closer to 100% in the current era. Furthermore, doctors are already using their phones in the workplace to improve their patient care; 59% of doctors said they used their phones to ascertain information from the internet and 30% of doctors said that they used work-related apps and E-books. This study highlighted that there is a potential demand for E-books that are intended for use by doctors in the workplace. There was discrepancy amongst the participants about whether they would use this E-book in an acute setting with the issue appearing to be the accessibility of the information and how the use of mobile phones would be perceived on the ward. Further research needs to be conducted on this subject to ascertain whether there is a need for an easily accessible app for students and doctors to use in the workplace.

However, the overall impression that emerges from the data is how personalizable a resource can be; a variety of different uses for the E-book have emerged from the range of participants. Personalized learning has been an approach that is a relatively recent phenomenon in educational implementation. It has been shown in the literature that students who personalized their own learning actually increase their level of motivation towards engaging in the subject content ( Duckworth *et al.*, 2009). Intrinsic motivation (IM) drives a person to pursue an activity due to curiosity, learning out of interest or enjoyment with no apparent reward except the activity itself ( Ryan and Deci, 2000; Dornan *et al.*, 2005; Kusurkar *et al.*, 2011). High IM is associated with better learning, better academic performance and achievement, better conceptual understanding, and higher levels of well-being than high extrinsic motivation ( Ten Cate, Kusurkar and Williams, 2011).

Self-determination theory (SDT) proposes that for IM to develop there is a requirement for fulfilment of three basic needs, namely: the need for autonomy, competence, and relatedness ( Kusurkar *et al.*, 2011; Ten Cate, Kusurkar and Williams, 2011). SDT puts forth autonomous motivation as a positive and therefore desired type of motivation leading to, amongst other positive outcomes; more deep learning **,** less superficial information processing, higher achievement, and enhanced well-being or adjustment ( Black and Deci, 2000; Vansteenkiste *et al.*, 2004, 2005; Soenens and Vansteenkiste, 2005; Kusurkar *et al.*, 2011).

As students develop autonomous personalized learning motivation increases. This increase in motivation with personalized learning has shown to increase the amount of time that students spend studying but also increases their satisfaction with working ( Prain *et al.*, 2013). It can also improve the student-student relationships and the student-teacher relationships as they aim to discover how each of their learning styles interacts with one another ( Edwards, 2005).

Personalization can also be used to support the use of co-design when developing resources such as an E-book. Co-design, whereby the user is engaged in the design process of the learning resource, it has been suggested, is a successful methodology insofar as it can support the satisfaction of the three basic psychological needs of SDT ( Dent-spargo, no date). It is therefore advocated that students are involved in the production of a learning resource ( Stradling and Saunders, 1993; Tomlinson and Kalbfleisch, 1998; Strong, Silver and Perini, 2001; Subban, 2006). This allows a level of personalization from the beginning so that students can identify before they use the E-book what sort of features would be useful to them in order to utilize the resource fully.

**Strengths of the study:** This study has allowed an insight into student uses of the E-book; reports from participants said that they felt that they could not give open feedback to University Faculty. Henceforth, this project allowed a greater insight because of the separation between the research team and the university faculty. Furthermore, conducting this research by using focus-groups allowed for ideas to be cultivated amongst the group, hence developing a greater depth of data. However, by keeping each group relatively small, it allowed every member of the group to be involved and to voice their opinion. This was an exploratory study which allowed the research team to obtain a large breadth of information to both improve this E-book but also to apply to future E-book development.

**Limitations of the study:** The PI’s relationship with participants could be seen as a limitation of the study. However, it is also a potential strength as participants may have been more in their discussions of the E-book because he was a peer. The second author (PC) and the project supervisor (AL) independently reviewed the transcripts to develop and alter the codes and themes to minimise this bias, neither had personal relationships with the participants. The PI assisted in the development of the E-book and the second author (PC) was the lead-clinician developing the educational package; however, the project supervisor wasn’t involved in the production of the E-book. This involvement in the production of the E-book could have introduced subconscious bias to desire that the e-Book being positively described. However, the involvement of the project supervisor in reviewing the codes resulted in a reduction in bias. This bias could have been minimised further by contracting an independent evaluator, however, this was not feasible as there was no budget for its creation or evaluation.

## Conclusion

In conclusion, this study used qualitative methods to identify themes related to the personalized use of an E-book within a medical curriculum. The personalisation was valued, and students found their own use of the resource, including using it for exams and teaching sessions, using it ‘on the go’ and using it in clinical practice. Educators should not fear personalization by students and acknowledge that learning resources may be used in ways beyond what they expected and that this finding is to be encouraged to benefit students.

## Take Home Messages

Educators should embrace, and not shy away from, the personalized use of a resource by their students; it is this autonomy that their students truly value.

## Notes On Contributors

**Dr Toby Price:** University of Leeds, UK, and Junior Doctor at Leeds Teaching Hospital Trust, Leeds, UK. https://orcid.org/0000-0002-4823-4848. Dr. Price gained a BSc in Medical Education and undertook this assessment as his final thesis.

**Dr. Peter Thomas Cartledge:** Honorary Lecturer at the University of Leeds, Leeds, UK and Associate Professor at Yale University (USA), Human Resources for Health, Rwanda.Dr. Cartledge has worked in medical education since 2004 and has been a full-time medical educator in Rwanda since 2016.
