# Supplementary material for: Discovering Students’ Personalised Uses of a Paediatrics Acute Care E-book – a Qualitative study
Source: MedEdPublish (2016). 2019 Oct 9;8:189. [Version 1] doi: 10.15694/mep.2019.000189.1 (PMC10712500; doi:10.15694/mep.2019.000189.1)
Supplement: Peer review history

# Reviewer report: Reviewer response for version 1

**DOI:** 10.21956/mep.19919.r30191

**Published:** 2019-11-17

**Authors:** Barbara Jennings

**Affiliations:** 1 Norwich Medical School

This review has been migrated. The reviewer awarded 4 stars out of 5 I found this to be an interesting and well-written article. It introduces us to qualitative research about how undergraduate medical students use a bespoke electronic (E-) book (pRRAPID), that the curriculum team has designed to support an acute care clinical attachment in paediatrics.The E-book is defined and critically appraised as a digital learning resource in the Introduction. The authors also establish the rationale for their study and describe the relevant curriculum supported by the E-book and cite the URL for this educational package: http://rrapid.leeds.ac.uk/ . The authors describe the use of focus groups in a very clear methods section. Only a small cohort of participants was recruited, and the data from 12 participants (3 focus groups) was analysed. It revealed five major ways that students used & personalised their E-book, including exam preparation and supporting clinical practice.In their discussion, the authors suggest study strengths and limitations, and they describe their research as “an exploratory study which allowed the research team to obtain a large breadth of information to both improve this E-book but also to apply to future E-book development”The article will be a useful and an interesting read for curriculum planners, teachers, and digital technologists who use or design E-books, or who curate digital resources for undergraduate medical students. Furthermore, the E-book is available as a free, open-access educational resource via the pRRAPID website.

Reviewer Expertise:

NA

No decision status is available

# Reviewer report: Reviewer response for version 1

**DOI:** 10.21956/mep.19919.r30192

**Published:** 2019-11-13

**Authors:** Trevor Gibbs

**Affiliations:** 1 AMEE

This review has been migrated. The reviewer awarded 4 stars out of 5 A very interesting paper written about topic ( e-learning or TEL) which is inviting much debate. I thought that this paper was well written and constructed. It covered a very important element of how students use and would prefer to use TEL.I would have hoped that more students would have involved themselves in this piece of research and one wonders why the numbers dropped so much. I would have also liked to have seen faculty involved in this- hat were their views as well as looking through a more negative lens- looking at the negative as well as the positive outcomes.I would recommend this paper to all involved in the use and development of this teaching and learning methodology.

Reviewer Expertise:

NA

No decision status is available
